# What the snake leaves in its wake: Functional limitations and disabilities among snakebite victims in Ghanaian communities

**DOI:** 10.1371/journal.pntd.0010322

**Published:** 2022-05-23

**Authors:** Leslie Mawuli Aglanu, John Humphrey Amuasi, Bob A. Schut, Jonathan Steinhorst, Alexis Beyuo, Chrisantus Danaah Dari, Melvin Katey Agbogbatey, Emmanuel Steve Blankson, Damien Punguyire, David G. Lalloo, Jörg Blessmann, Kabiru Mohammed Abass, Robert A. Harrison, Ymkje Stienstra

**Affiliations:** 1 University of Groningen, Department of Internal Medicine/Infectious Diseases, University Medical Centre Groningen, Groningen, The Netherlands; 2 Global Health and Infectious Diseases Research Group, Kumasi Centre for Collaborative Research in Tropical Medicine, Kumasi, Ghana; 3 Department of Global Health, School of Public Health, Kwame Nkrumah University of Science and Technology, Kumasi, Ghana; 4 Department of Development Studies, Simon Diedong Dombo University of Business and Integrated Development Studies, Upper West Region, Wa, Ghana; 5 Regional Health Directorate, Ghana Health Service, Upper West Region, Wa, Ghana; 6 Department of Emergency Medicine, Upper West Regional Hospital, Wa, Ghana; 7 Centre for Snakebite Research and Interventions, Liverpool School of Tropical Medicine, Liverpool, United Kingdom; 8 Bernhard Nocht Institute for Tropical Medicine, Department of Implementation Research, Hamburg, Germany; 9 Presbyterian Hospital, Agogo, Ghana; College of Health Sciences, Bayero University Kano, NIGERIA

## Abstract

**Background:**

The estimated five million snakebites per year are an important health problem that mainly affect rural poor populations. The global goal is to halve both mortality and morbidity from this neglected tropical disease by 2030. Data on snakebite morbidity are sparse and mainly obtained from hospital records.

**Methods:**

This community-based study was conducted among 379 rural residents with or without a history of snakebite in the Ashanti and Upper West regions of Ghana. All participants in the snakebite group were bitten at least six months before the day of survey. The World Health Organisation Disability Assessment Schedule 2.0 (WHODAS 2.0) and the Buruli Ulcer Functional Limitation Score were used to obtain patient-reported measure of functioning and disability. Long-term consequences were evaluated based on the severity of the symptoms at the time of the snakebite.

**Findings:**

The median (IQR) time since the snakebite was 8.0 (3.5–16.5) years. The relative risk of disability was 1.54 (95% CI, 1.17–2.03) in the snakebite group compared to the community controls. Among patients with clinical symptoms suggesting envenoming at the time of bite, 35% had mild/moderate disabilities compared to 20% in the control group. The disability domains mainly affected by snakebite envenoming were cognition level, mobility, life activities and participation in society. A combination of the severity of symptoms at the time of the bite, age, gender and region of residence most accurately predicted the odds of having functional limitations and disabilities.

**Conclusion:**

The burden of snakebite in the community includes long-term disabilities of mild to moderate severity, which need to be considered when designing appropriate public health interventions. Estimating the total burden of snakebite is complicated by geographic differences in types of snakes and their clinical manifestations.

## Author summary

Snakebite is a neglected tropical disease of public health concern. The majority of cases occur in tropical rural regions where access to adequate medical interventions is limited. The outcome of these bites may cause a range of manifestations, varying from local lesions to life threatening effects and death. Available estimates of snakebite burden are often based on severe complications such as death and amputations and are limited to health facility-based evaluations. We conducted a community-based study to identify the long-term consequences of snakebite by comparing participants with or without a history of snakebite in two regions in Ghana. A history of snakebite was associated with the chance of having disabilities. Combining community estimates of mild to moderate complications with data on severe complications from the health system level provides a more comprehensive overview of the overall snakebite burden. The incorporation of mental health and rehabilitation interventions into integrated neglected tropical diseases (NTD) programmes can reduce the risk of disabilities and improve the wellbeing of snakebite and other NTD patients.

## Introduction

Despite the recent recognition and renewed commitment by the global neglected tropical diseases (NTD) community, snakebite continues to afflict millions annually [1–5]. The risk of human-snake conflict is highest in poor rural regions in the tropics due to the overlapping habitat and the predominantly high degree of labour-intensive agrarian economic activities prevailing in these regions [6, 7]. Of the over 3,000 species of snakes known globally, more than 600 belonging to the families of Viperidae, Elapidae and Atractaspidae are venomous [7, 8]. Based on publications on the epidemiology and clinical manifestations of snakebites, the medically important snake species in Ghana include the West African carpet viper (*Echis ocellatus)*, Puff adder (*Bitis arietans)*, Black-necked spitting cobra (*Naja nigricollis)*, West African green mamba (*Dendroaspis viridis)* and Causus species such as the night adder and the spotted night adder [9, 10]. Effects of the bites from these snakes can vary from local lesions to life threatening effects and death [11–15]. In Sub-Sahara Africa, snakebite envenoming is estimated to cause over 7,300 deaths and up to 14,600 amputations annually [16]. Based on national health system records, the incidence of snakebite in Ghana is estimated at 35/100,000 persons per year while a community-based prevalence of having a history of snakebite was estimated to be 6% with a case fatality rate of 3% in northern Ghana [9]. At the health facility level, an 11% case fatality rate was reported in a pre-intervention study at a rural health facility in the central part of the country [17]. Complications such as haematoma, impairment and even loss of vision, intracerebral haemorrhage, compartment syndrome and amputation have been reported as some of the acute consequences of snakebite envenoming in Ghana [18–20]. Appropriate healthcare seeking behaviour and adequate health staff competence in diagnosis and management protocol compliance have however proven to significantly reduce the mortality rate snakebite [17].

Generally, the long-term functional limitations and disabilities of snakebite have received little attention [12, 21–23]. In Ghana, the disability adjusted life years (DALY) caused by snakebite, calculated as the number of snakebite envenoming related deaths and the years of life lived with amputations with a disability weight of 0.13, was estimated at 22,243 [20]. All of the sparsely available data on the long-term consequences of snakebite is health facility-based despite evidence of a significant proportion of victims never reporting to health facilities [24–26]. In many rural communities, snakebites have varying connotations arising from spiritual/mythical or natural interpretations [26]. These implications, along with financial constraints and lack of availability and access to antivenoms influence decisions on the first point-of-care. These perceptions and constraints contribute to the acute outcomes registered in health facilities, while the outcomes and consequences for those who do not visit any health facility remain unaccounted for.

To better comprehend the burden of snakebites on rural communities in Ghana, we looked at functional limitations and disabilities in community members with and without a history of snakebite. We described the relative risk of functional limitations and disabilities and the factors best predicting these long-term consequences. Furthermore, the type of limitations and their severity were evaluated based on the likelihood of envenoming deduced from the symptoms after the bite.

## Methods

### Ethics statement

Permission to conduct the study was obtained from the Regional Health Directorate of the Upper West Region, with the Ghana Health Service Ethics Review Committee and the Medical Ethical Committee of the University Medical Centre Groningen, The Netherlands (GHS-ERC010/03/20, NL-LTc201900047) providing ethical approvals. Signed or thumb printed informed consent was obtained from all participants after the content of the information sheet and consent forms were read and translated into Dagaare, Waale or Twi depending on the preferred language of the participant. Assent was obtained from children and consent from their parents or guardians before their inclusion in the study. The community health volunteers acted as witnesses in the consent processes.

### Study population and sampling

**Fig 1:**
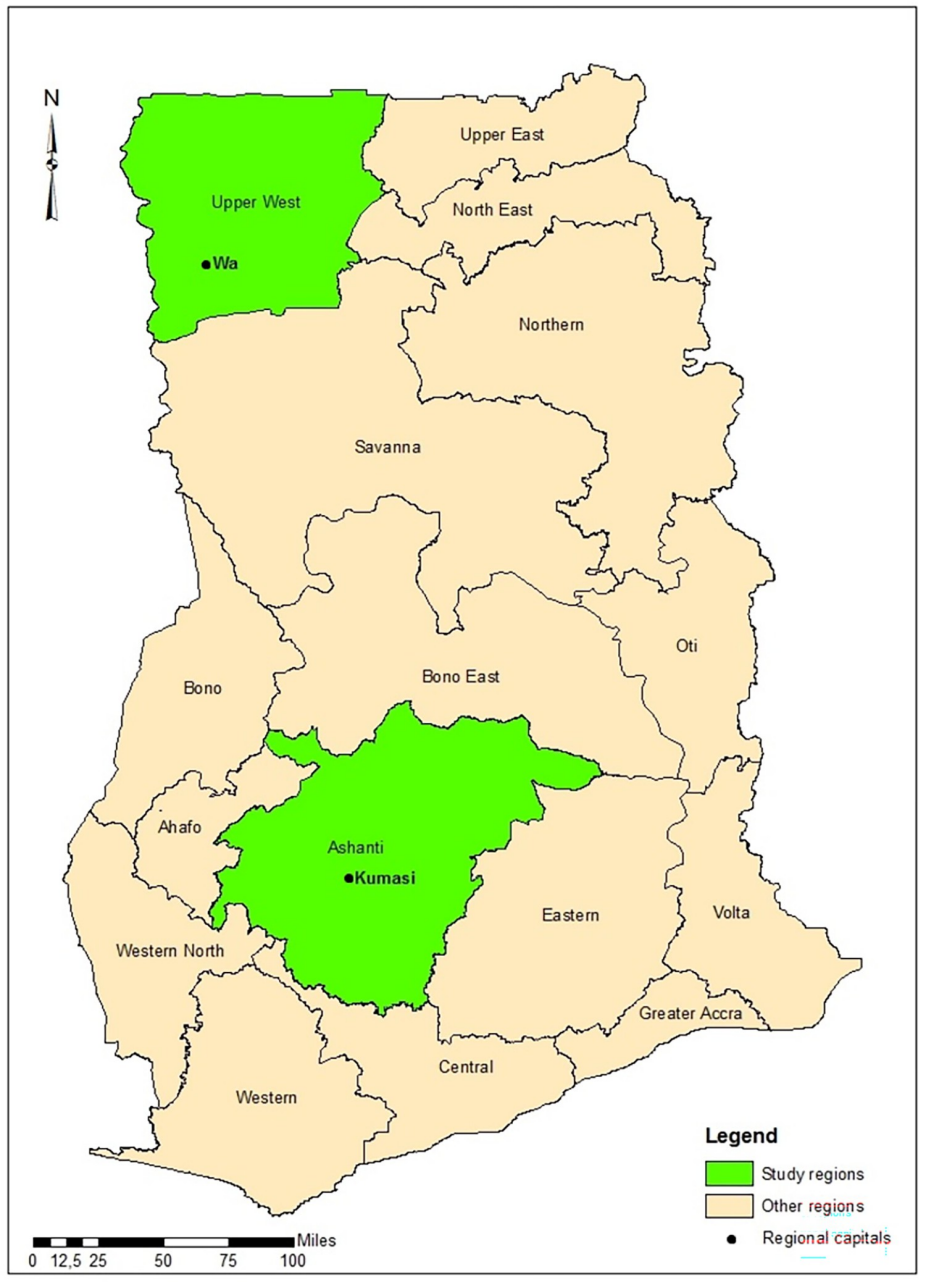
A map of Ghana showing the study regions (ArcGIS software version 10.8.1, ESRI Inc., Redlands, CA, U.S.A).

Data for this cross-sectional cohort study was collected in the Ashanti and the Upper West regions of Ghana in March 2020 and March 2021, respectively. The timing of the data collection was influenced by COVID-19 travel restriction. Based on national health facility-reported data on snakebite from 2015 to 2019, the Ashanti and Upper West regions reported the highest annual case records of snakebite in the country. These two regions are located at different parts of the country and have distinct climate and socioeconomic characteristics (Fig 1). According to the 2021 Population and Housing Census (PHC), the Ashanti region and the Upper West regions have a population of 5,432,485 and 904,695 respectively [27].

We visited the Wa Municipal Hospital in the Upper West region and the Agogo Presbyterian Hospital in the Ashanti region; both of which are among the highest health facilities reporting snakebite cases to the national surveillance system. From the snakebite records of these hospitals we selected five high reporting communities in the catchment of each hospital. Sampling in the Ashanti region was however affected by the COVID-19 restrictions. Data collection was carried out by an experienced team of researchers including health workers. The team was trained on the data collection tools and were provided with visual aids to support in the identification of snake species. With support from community health volunteers, we used a snowball sampling approach to identify all individuals with a history of snakebite in these closely knit communities. To ensure the detection of long-term consequences after snakebite, only victims whose snakebite incident occurred at least six months before the day of survey were eligible for inclusion. Due to the nature of the questionnaire and reliance on patient history, children below 10 years of age and participants who could not recall and narrate the snakebite incident were excluded. Control subjects from the community without a history of snakebite were recruited using random sampling from households in the same communities. We randomly selected these control subjects from households by picking a direction from a bag containing all the 16 cardinal directions inscribed on separate pieces of paper. Control subjects were matched to victims by the communities and by an age interval of 10 years. Assuming an alpha value of 0.05, a study power of 80%, an estimated functional limitation and disability prevalence of 3% based on data collected during the 2010 PHC in the general population [28], we aimed to include a minimum of 183 participants in each group to detect a four-fold higher prevalence of disabilities in the snakebite victims than in the control group.

### Participant assessment and questionnaires

Sociodemographic data such as age, sex, occupation, education and general medical history were collected to describe the structure and social characteristics of the study population. Data on participant reported long-term impairment, sequela and impaired joint function were collected to assess disability. Characteristics such as number of bites experienced, location of the bite, symptoms of envenoming, species of snake responsible for the bite identified using victims’ description and visual aids, and the type of medical care received were recorded.

Participants were assigned into three different groups. Snakebite envenoming (SBE)—participants who reported multiple clinical symptoms (redness, swelling around the bite site, haematoma and bleeding for more than an hour) were classified as snakebite envenoming

Snakebite (SB)—participants who reported no symptoms at all or only localised redness or swelling, possibly as a result of dry bites or bites by non-venomous snakes

Controls–participants without any history of snakebite. This approach limited mistaking trauma triggered responses as symptoms of envenoming [29].

The World Health Organisation Disability Assessment Schedule (WHODAS 2.0) was used to obtain a patient-reported measure of functioning and disability. WHODAS 2.0 has been used cross-culturally and across different diseases and disease-related states. The questionnaire has six domains (cognition, mobility, self-care, relationships, life activities and participation in society) with a five-point Likert-scale response for each item. We used the 12+24-item version and the item-response-theory based scoring method, which takes into account multiple levels of difficulty for each item for scoring [30]. The domain and general disability scores were transformed into a metric ranging from zero to 100 using the SPSS syntax for automatic computation provided in the manual. A score of zero indicates no disability and a higher score indicates more disability. Comparison with other recognized disability measurement instruments has shown a good concurrent validity in patient classification; conformity to Rasch scaling properties across populations, and good responsiveness [31]. In assessing the severity of disability, the WHODAS 2.0 output were categorised into the International Classification of Functioning, Disability and Health (ICF) disability categories: no disability (0–4%), mild disability (5–24%), moderate disability (25–49%), severe disability (50–95%) and extreme disability (96–100%). The instrument has been used in over 27 areas of research in over 94 countries and has been described as a generally valid and reliable instrument for assessing various forms of disability [30–35].

We used the Buruli Ulcer Functional Limitation Score (BUFLS) to further characterise the type of functional limitations. Even though the BUFLS was developed to assess functional limitations in Buruli Ulcer patients, we evaluate its validity and applicability in snakebite patients by comparing its outcome to that of the WHODAS 2.0. The 19-item BUFLS questionnaire has been used in countries in West Africa. Its validity has been established by correlations with range of motion and the global impression of functional limitation scale [36]. The 19-item BUFLS questionnaire could provide more details on the type of activities limited. The questionnaire is categorised into food preparation, personal care, daily work activities and mobility domains. The response to each item was scored using an ordinal scale. If the activity could be performed without difficulties and on a level comparable to other community members of the same sex and age, zero points were allotted. An activity that could be performed but with difficulties was graded with one point and activities that could not be performed at all were marked with two points. If the item was not valid for the respondent, e.g., the person was too young or old, the item was scored as ‘not applicable’ [36, 37]. To calculate the functional limitation score, the sum of the scores was divided by the maximal score applicable for the participant and multiplied by 100. The final score ranged from zero to 100 where a zero meant no limitation and higher scores indicated maximum functional limitation. The functional limitation score was not calculated for those with more than six items of the questionnaire not being applicable.

### Data analysis

The data was collected in REDCap and analysed using IBM SPSS Statistics for Windows version 26. Descriptive statistics were used to describe the demographic characteristics and participant functional limitations. Relative risk (RR) at 95% confidence intervals (CI) were calculated to estimate the effect size of snakebite-induced disabilities. We conducted a dichotomous logistic regression analysis with manual backward selection based on P-values less than 0.05 to identify the set of variables that best predicted functional limitations and disabilities. Gender, region, level of education, occupation and severity of the bite were marked as categorical covariates. All these variables, but severity of the bite, have been identified in literature as major predisposing factors of snakebite. We assessed the variance of the model using Nagelkerke R squared and determined the goodness-of-fit by the Hosmer-Lemeshow test.

## Results

A total of 379 participants with a median age of 37 (26–53) were included as snakebite victims or community controls. The median years since the snakebite event was 8.0 (3.5–16.5). Snakebite victims accounted for 193 (50.9%) of the participants with females making up for 198 (52.2%) of the total participants recruited (Table 1). Over 80% of the study participants in each group were recruited in the Upper West region, partially as a consequence of COVID-19 travel restrictions. The level of education differed between the two groups. In the control group, more participants had attained secondary or tertiary level education. The distribution of the type of occupation was similar in both groups.

**Table 1:** Characteristics of the study participants (*N* = 379).

		Snakebite Victims n (%)	Controls n (%)	P-value
**Demographics and Functional limitations**		193 (50.9)	186 (49.1)	
**Region**	Ashanti Upper West	36 (18.7) 157 (81.3)	20 (10.8) 166 (89.2)	0.030*
**Gender**	Male Female	95 (49.2) 98 (50.8)	86 (46.2) 100 (53.8)	0.561*
**Age** (years)	Median (IQR)	37 (26.5–52.0)	36 (26.0–53.5)	0.896^Ϫ^
**Level of education**	No education Primary education Secondary education Tertiary education	112 (58.0) 53 (27.5) 25 (13.0) 3 (1.6)	99 (53.2) 33 (17.7) 34 (18.3) 20 (10.8)	**0.003** ^Φ^
**Occupation**	Farmer Trader Other Student/Not employed	131 (67.9) 15 (7.8) 14 (7.3) 33 (17.1)	108 (58.1) 18 (9.7) 24 (12.9) 36 (19.4)	0.163*
**Observed sequela** ^**⸭**^	No Yes	157 (81.3) 36 (18.7)	177 (95.2) 9 (4.8)	**<0.001** *
**Type of sequela observed**	Amputation Scar tissue Joint deformities Vision impairment Hemiparesis Swelling/oedema Other Multiple signs No sequela	3 (1.6) 17 (8.8) 7 (3.6) 0 (0.0) 1 (0.5) 2 (1.0) 2 (1.0) 4 (2.1) 157 (81.3)	0 (0.0) 0 (0.0) 2 (1.1) 2 (1.1) 1 (0.5) 1 (0.5) 3 (1.6) 0 (0.0) 177 (95.2)	**<0.001** ^**ϰ**^
**Participant reported impaired joint function**	Back Shoulders Wrist Hand/fingers Knee Ankle Multiple joints No joint impairment	0 (0.0) 0 (0.0) 3 (1.6) 10 (5.2) 1 (0.5) 5 (2.6) 0 (0.0) 174 (90.2)	1 (0.5) 1 (0.5) 0 (0.0) 1 (0.5) 0 (0.0) 1 (0.5) 2 (1.1) 180 (96.8)	**0.001** ^**ϰ**^

* Pearson Chi-Square test

^Ϫ^ Mann–Whitney U test

^Φ^ Linear-by-Linear Association

**^ϰ^** Fisher’s Exact test

**^⸭^**Observed sequelae due to snakebite or earlier disease or trauma

Out of the 193 snakebite victims, 161 (83.4%) had experienced one and 32 (16.6%) had experienced two or more snakebites. A total of 125 (64.8%) of the 193 victims saw the snake responsible for the bite. Out of this group, 61 (48.8%) described it as *Echis ocellatus*, 24 (19.2%) as *Naja nigricollis* and 11 (8.8%) as *Bitis arietans*. One person described a *Naja* species and another *Dendroaspis viridis*. A total of 19 (15.2%) participants attributed the bite to snakes known to be non-venomous and 8 (6.4%) could not adequately describe the snake responsible for the bite. Based on the symptoms enumerated by the snakebite victims approximately 64% had dry bites or bites by non-venomous snakes.

Approximately 41% (79 out of 193) of the snakebite victims sought treatment from traditional healers only, 111 (57.5%) sought treatment from a health facility only or from both a health facility and a traditional healer, and three (1.6%) used home remedies. Out of the 69 participants who reported multiple clinical symptoms suggesting envenoming, 25 (36.2%) sought treatment from a traditional healer only. In comparison, out of the 124 participants who reported no symptoms at all or only localised redness or swelling, dry bites or bites by non-venomous snake, 54 (43.5%) sought treatment exclusively from a traditional healer. Health-seeking behaviour was not associated with the level of education. Of those who sought treatment from a health facility, about 78% (87 out of 111) indicated that they received anitvenom. Approximately 17% could not recall whether they received antivenom as part of the treatment or not.

**Fig 2:**
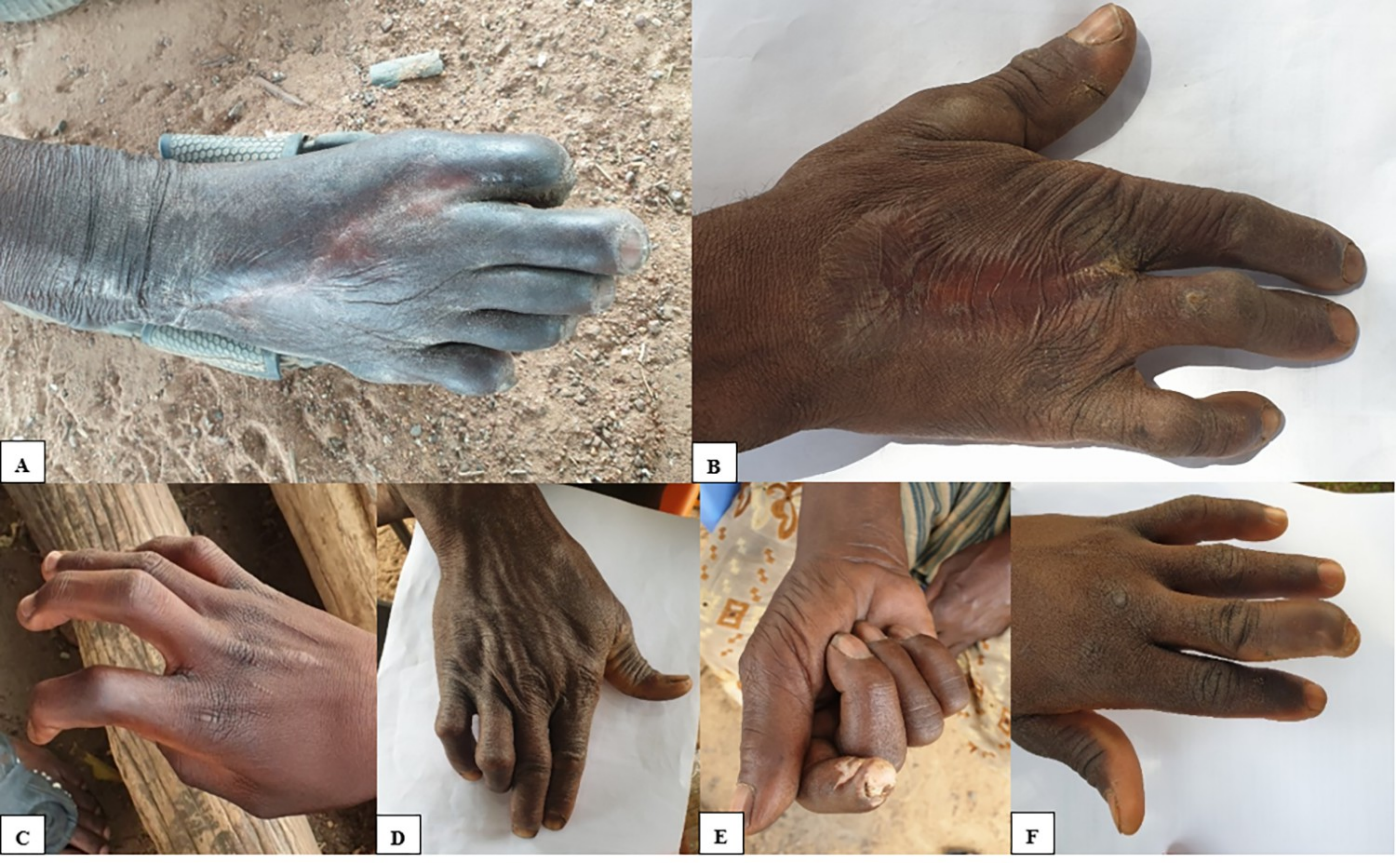
Various localized residual effects of snakebite. A: Amputation at the proximal interphalangeal joint of the big toe (Echis ocellatus reported). B: Amputation of the middle finger (Naja nigricollis reported). C: Contracture deformity of the distal interphalangeal joint of the index finger (Bitis arietans reported). D: Contracture deformity of the proximal and distal interphalangeal joints of the ring and little fingers (Echis ocellatus reported). E: Contracture deformity of the proximal and distal interphalangeal joints of the index finger with a chronic ulcer (snake not seen). F: Contracture deformity of the proximal interphalangeal joint of the middle finger (Bitis arietans reported). This participant did not report to a health facility for treatment. Participants in images A and E reported mild disabilities based on the WHODAS 2.0 total scores. All the other participants reported no disability.

Scar tissue and joint impairment were the main sequelae of snakebites in the study population (Fig 2). The type of sequela observed in participants with multiple signs of sequelae include amputation, scar tissue, swelling/oedema and vision impairment. The majority of the snakebite victims (83.3%) with sequelae directly attributed it to the snakebite. However, the victim with hemiparesis did not attribute his disability to the snakebite. In total, four (2%) snakebite related amputations were recorded. Compared to the community controls, the snakebite victims had a higher risk of an impaired joint function (9.8% vs. 3.2%; RR, 3.05; 95% CI, 1.25–7.47).

Using the WHODAS 2.0 scores >0, the snakebite victims group recorded higher risk of having functional limitation and disabilities in comparison to the community controls (44.8% vs. 29.0%; RR, 1.54; 95% CI, 1.17–2.03).

**Fig 3:**
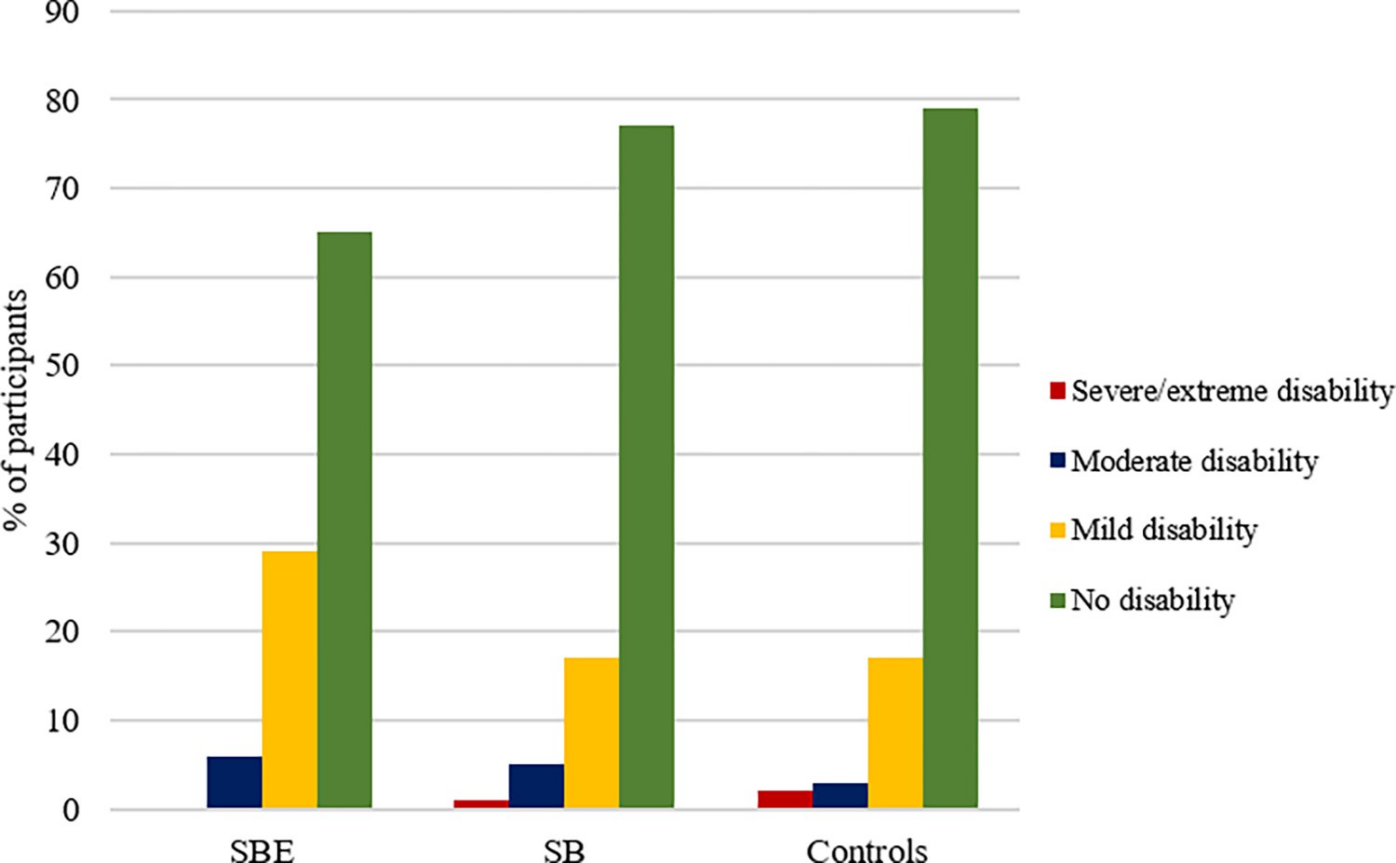
Severity of disability based on the WHODAS 2.0 ICF categories shown for the study.

**Fig 4:**
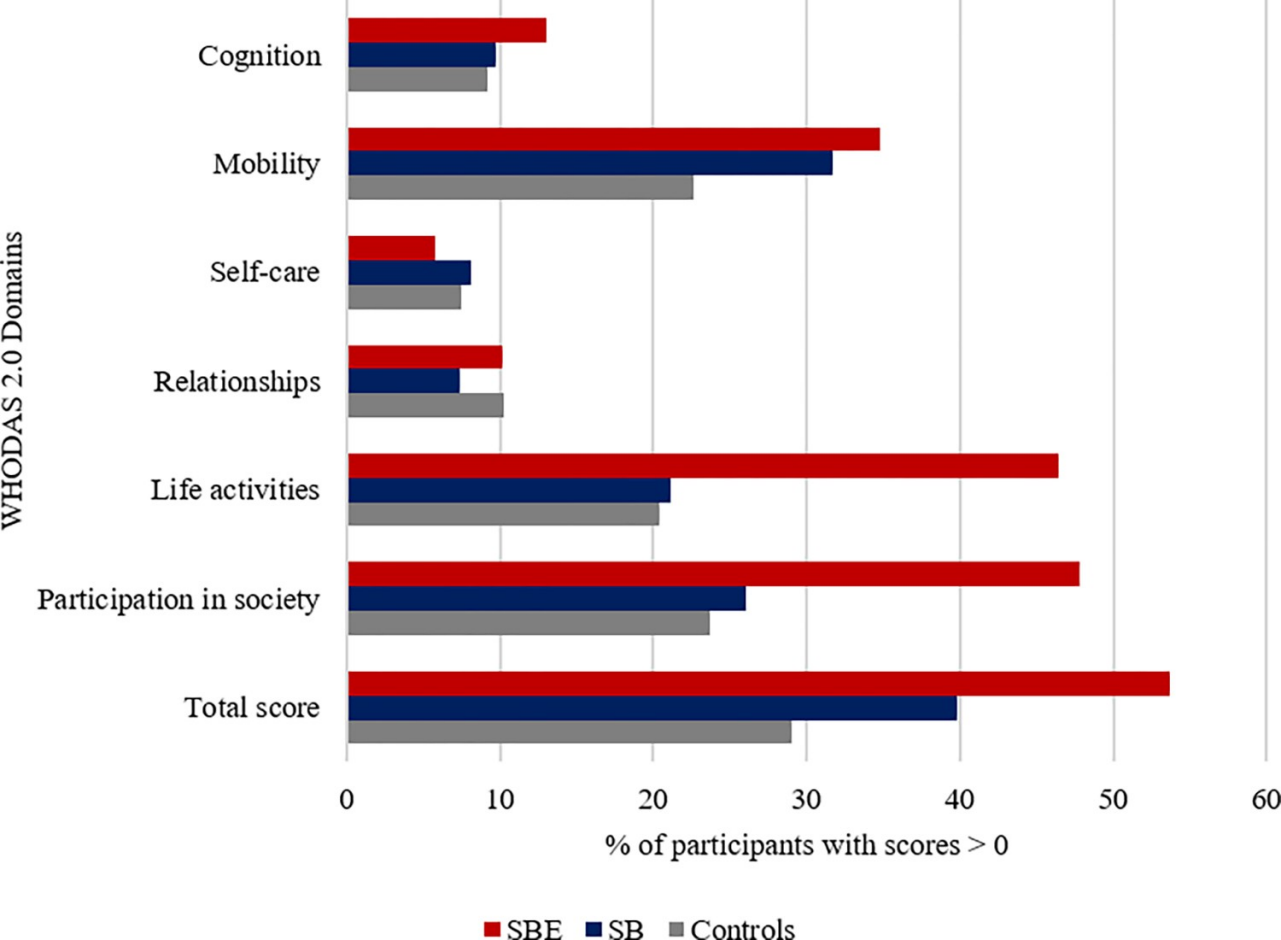
Participants in each study group with perceived disabilities present per WHODAS 2.0 domain scores.

Among the snakebite victims group, we analysed the severity of functional limitations and disabilities in victims based on whether they reported multiple clinical symptoms suggesting snakebite envenoming (SBE), or only localised redness or swelling around the bite site or no symptom at all, suggesting dry or non-venomous snakebites (SB). Approximately 36% of the snakebite victims had symptoms suggesting SBE. Fig 3 shows that victims of the SBE subgroup recorded a higher proportion of mild and moderate disabilities relative to both the SB subgroup and the control group. There was no difference between the SB subgroup and the control group. Within the domains of the WHODAS 2.0, a higher percentage of the participants in the SBE subgroup recorded functional limitations and disabilities in cognition, mobility, life activities, and participation in society in comparison to the SB subgroup and community control group (Fig 4).

Comparing the proportions of participants with positive scores (> 0) in the SBE and SB subgroups to the community control groups, we found the SBE group had higher risk of having functional limitations and disabilities (Table 2).

**Table 2:** WHODAS 2.0 assessment of the study participants.

		SBE n (%)	RR SBE*Control	95% CI	SB n (%)	RR SB*Control	95% CI	Controls n (%)
Measure of functional limitation and disability		69 (18.2)			124 (32.7)			186 (49.1)
WHODAS 2.0	Score > 0	37 (53.6)	1.85	1.35–2.53	49 (39.8)	1.37	1.00–1.88	54 (29.0)
	Score = 0	32 (46.4)			74 (60.2)			132 (71.0)
	Not applicable	0 (0.0)			1 (0.8)			0 (0.0)
WHODAS 2.0	Median (IQR)	0.9 (0.0–9.0)	-	-	0.0 (0.0–4.7)	-	-	0.0 (0.0–3.1)

WHODAS 2.0: World Health Organisation Disability Assessment Schedule; SBE: Snakebite envenoming; SB: dry or non-venomous bite; RR: Relative risk; 95% CI: 95% confidence interval of relative risks

As measured by the BUFLS, no difference was found in the risk of having functional limitations between the SBE subgroup and the community controls (47.8% vs. 39.8%; RR, 1.20; 95% CI, 0.88–1.63). A similar result was found between the SB subgroup and the community controls (34.7% vs. 39.8%; RR, 0.87; 95% CI, 0.65–1.18).

Based on the WHODAS 2.0 and using variables measured in this study, the most appropriate model predicting disability in the snakebite population included the variables age, gender and region of residence (Nagelkerke R^2^ = 0.17, Hosmer-Lemeshow goodness-of-fit test P = 0.59, Table 3). Gender and region of residence produced a higher effect on the model.

**Table 3:** Results of the dichotomous logistic regression analysis on the variables best predicting disabilities in snakebite victims.

Predictors	OR	95% CI
**Severity of the snakebite** ** SB** ** SBE**	- 1.91	1.01–3.62
Age (years)	1.03	1.01–1.04
**Gender** ** Male** ** Female**	- 3.13	1.66–5.89
**Region** ** Upper West** ** Ashanti**	- 2.74	1.22–6.14

OR: Odds ratio; SB: Dry or non-venomous snakebite; SBE: Snakebite envenoming; Nagelkerke R^2^ = 0.169; 95% CI: 95% confidence interval of odds ratios

There was no difference in the risk of having disabilities among victims of the SBE subgroup if they decided to visit the hospital versus visiting a traditional healer only (56.8% vs 48.0%; RR, 1.18; 95% CI, 0.73–1.92).

## Discussion

The ease with which snakebite victims were identified in these communities during the data collection suggests frequent interactions between humans and snakes, despite limiting the inclusion criteria to victims whose history of snakebite was at least 6 months old on the day of survey. We found a history of snakebite to be associated with the presence of disabilities. The risk of impaired joint function among the snakebite victims was three times higher than the risk among the community controls. The risk of disabilities among the snakebite victims who reported multiple clinical symptoms suggesting snakebite envenoming (SBE) was 1.85 times higher than the risk in the community controls, with 54% of the SBE victims recording some form of disability compared to 29% in the community controls. The disability domains mainly affected by snakebite envenoming are cognition level on understanding and communicating, mobility, participation in society, and life activities such as undertaking domestic, work or school activities. Victims of dry bites or bites from non-venomous snakes seem to have no long-term consequences due to the snakebite. In contrast to the WHODAS 2.0, the BUFLS did not show any difference in functional limitations between the snakebite victims and the community controls even though both are patient-reported outcome measures. As an instrument developed to detect mainly severe functional limitations, the results in this study reflects the BUFLS’ low sensitivity to mild/moderate functional limitations as found in another study [38].

The burden of snakebite is multidimensional, making estimation of the true burden a major challenge. In Sub-Sahara Africa, a meta-analysis on the burden of snakebite found 6% of victims had sequelae such as contractures, vision impairment, swellings and scars, and an amputation rate of 3% [39]. However, most of the studies included in this meta-analysis sourced data from national health reporting systems and hospital records which does not necessarily represent the total burden of SBE in the communities. Active evaluation of long-term consequences in the community leads to a higher burden as detected in publications from Sri Lanka. A very diverse pattern of long-term physical consequences of snakebite was reported in communities in Sri Lanka based on a screening questionnaire followed by a medical evaluation if positive. A validated tool to assess disabilities was not included and community members without a snakebite history were not evaluated [21]. In another study, a high percentage of ongoing psychological morbidity was found in snakebite victims between 1 and 3 years after the bite compared with community controls. The Sheehan disability scale, a validated tool to measure functional impairment associated with anxiety disorders, indicated 14% (versus 3% in the controls) had such a functional impairment [40]. Fifteen out of the 88 (17%) snakebite victims furthermore reported residual physical disabilities. In Nigeria, persisting physical disabilities were observed in 16 (11.4%) out of the 140 snakebite victims identified from health facility records [41]. In a retrospective cohort study on snakebite wound management using negative pressure wound therapy (NPWT), necrosis and infection rates were 36.8% and 4.3%, and 13.2% and 4.3% in the non-NPWT and NPWT groups respectively [42]. This finding emphasise that appropriate wound management is essential in reducing further complications such as scars, amputations and range of motion impairments. The high prevalence of residual physical sequelae in our study stress that more effort is needed to promote good wound management both at the communality level and within the healthcare system. Using the Post-Traumatic Checklist (PCL-C) in the Nigerian study, 43% of the snakebite victims compared to 28% of their matched relatives with no history of snakebite showed post-traumatic stress disorder. These data together emphasize the multifaceted nature of the burden of snakebite. To assess the true burden, mild and moderate consequences need to be included in the total burden estimation. Furthermore, health systems data ought to be complemented with community-level data for a more accurate estimation of the exact population burden.

Within the six domains of the WHODAS 2.0, a higher proportion of the snakebite envenoming victims had disabilities in the level of cognition, understanding and communicating, mobility, participating in community activities and life activities such as undertaking domestic, work or school responsibilities. Snakebite had impact on the same WHODAS 2.0 domains among 58 snakebite victims in rural Amazonian communities in Brazil [43]. In a semi-urban region in Nigeria, a history of snakebite was associated with poor quality of life in psychological and social domains of the World Health Organization Quality of Life (WHOQOL)-BREF [41]. Among 48 patients with skin neglected tropical diseases (NTDs) in Nigeria, the 12-item WHODAS instrument also showed a higher disease burden on the level of cognition, mobility, life activities, and participation in society domains [44]. Aside from the physical burden, these findings highlight similar mental health and psychosocial challenges of snakebite victims to victims of other NTDs. Acknowledging the high risk of mental health conditions, the WHO advocates for the use of psychosocial and rehabilitation interventions to holistically address the health needs of NTD victims [45, 46]. Findings from a brief psychosocial intervention for snakebite victims and self-care intervention for other NTDs victims have proved effective in reducing psychiatric symptoms and disability, and improving disease impairment status among patients [44, 47]. These findings suggest that NTDs with similar disease pattern and effects can benefit from strong collaborative and integrated interventions which can be implemented as part of first lines of care.

Our finding shows a combination of the severity of the bite, age, gender and region of residence most accurately predicted the odds of having disabilities. Increased age and the female gender contributed to the chance of having disabilities. A similar pattern has been reported in other studies despite males often reporting higher number of snakebite cases [17, 21]. This outcome could be attributed to the predisposing socioeconomic conditions and the daily responsibilities of females in rural communities. Apart from gender and age, the region of residence contributed to the chance of disabilities. This may be related to the locally prevalent snake species. In the Ashanti region, cytotoxic species such as *Naja nigricollis* and *Bitis arietans* are more common and their victims more likely to develop impaired joint function as compared to bite victims of *Echis ocellatus*, which is more common in the Upper West region and associated with bites primarily causing clotting impairment and death. The risk of clotting impairment and death were however not included in this study. This geographical difference illustrates that there may be important differences within countries that may require different interventions to prevent long-term consequences (e.g. needs for physical therapy or mental health support) depending on the type of snakes prevalent. It also illustrates that total population disability estimations using DALYs should be based on data obtained from different regions with different snakes prevalent to be reliable. Dry bites or bites from non-venomous snakes in our study did not contribute to long-term disabilities as assessed.

We decided not to produce DALY estimates since the severity of bites and the regional differences found suggested that the impact of a snakebite depended on the characteristics of the snake species. Furthermore, we did not include data on victims who died from snakebite due to the high chance of multiple counting at the community level. Additionally, the WHODAS 2.0 results in the control group further complicate a valid DALY estimate. We advocate for a specific disability weight for snakebite envenoming which can be used when calculating the global burden of snakebite, but this would need to take into account the clinically diverse impact of different snakes in different regions.

The inclusion of community members provided data from victims often excluded in burden estimates. The WHODAS 2.0 results from the community controls clearly illustrate the need to include controls when studying the burden of snakebites. We considered the different domains in the WHODAS 2.0 for a more holistic approach to evaluating long-term consequences than the mere counting of amputations and deaths. Furthermore, the WHODAS 2.0 can be used regardless of the snakes responsible for the bite. Nevertheless, we think specific mental health consequences such as anxiety disorders or mood disorders need specific attention in the future.

The study had limitations. The retrospective approach used in assessing participants’ snakebite history, makes the interpretation of the data susceptible to recall bias. We also relied on the clinical symptoms at the time of the bite as reported by the participants to distinguish between dry or non-venomous bites and venomous bites. In addition, the study was conducted in the two regions reported to have the highest annual case records of snakebite in the country. Geographical differences limit the options to extrapolate our findings to the national or international estimates. To better understand the population-based long-term consequences of snakebite, further research should include multiple regions and ideally analyse the burden per type of bite using a syndromic approach. Our sample size calculation was based on the 3% national disability estimate. Compared to the “first” national disability estimate based on data collected during the 2010 PHC, and the recent 2021 PHC we found a considerably different prevalence even in the community controls using the WHODAS 2.0. The difference could be attributed to how the data was collected. During the Census, individuals were simply asked if they had any serious disability that limit full participation in life activities such as mobility, work and social life. The national estimate is therefore likely biased towards severe physical disabilities. Since none of the snakebite envenoming victims in our study reported severe disabilities, the use of snowballing as the sampling approach was not biased to victims with overt disabilities.

## Conclusion

To better appreciate the true magnitude of the burden of snakebite envenoming, subsequent assessments of the disease burden should include mild and moderate long-term consequences along with the more severe consequences of amputation and death. These mild to moderately severe disabilities are not included in hospital-based estimates of snakebite burden and also occur in victims who do not visit hospitals. Community members with possible dry bites or bites from non-venomous snakes showed no long-term consequences in our study. Estimating the total burden of snakebites is complicated by geographic variations in types of snakes and their clinical manifestations. Assessing a national burden of snakebites therefore requires careful planning in multiple regions. Attention to non-acute pathological effects of snakebite, mental health and the establishment of rehabilitation programmes could reduce the risk of disabilities and improve the quality of life of snakebite victims.

## References

[1] Longbottom J, Shearer FM, Devine M, Alcoba G, Chappuis F, Weiss DJ, et al. Vulnerability to snakebite envenoming: a global mapping of hotspots. The Lancet 2018;392(10148):673–84 doi: 10.1016/S0140-6736(18)31224-8 30017551 PMC6115328

[2] Malecela MN, Ducker C. A road map for neglected tropical diseases 2021–2030. Transactions of the Royal Society of Tropical Medicine and Hygiene. 2021;115(2):121–3. doi: 10.1093/trstmh/trab002 33508095 PMC7842088

[3] WHO. Ending the neglect to attain the sustainable development goals: a road map for neglected tropical diseases 2021–2030. World Health Organization, 2020 ISBN: 978 92 4 001035 2. Available from https://www.who.int/publications/i/item/9789240010352.

[4] Williams HF, Layfield HJ, Vallance T, Patel K, Bicknell AB, Trim SA, et al. The Urgent Need to Develop Novel Strategies for the Diagnosis and Treatment of Snakebites. Toxins. 2019;11(6):363. doi: 10.3390/toxins11060363 31226842 PMC6628419

[5] Harrison RA, Casewell NR, Ainsworth SA, Lalloo DG. The time is now: a call for action to translate recent momentum on tackling tropical snakebite into sustained benefit for victims. Transactions of the Royal Society of Tropical Medicine and Hygiene 2019;113(12):835–8. doi: 10.1093/trstmh/try134 30668842 PMC6903789

[6] Chippaux J-P. Snakebite envenomation turns again into a neglected tropical disease! Journal of Venomous Animals and Toxins including Tropical Diseases. 2017;23(1):1–2. doi: 10.1186/s40409-017-0127-6 28804495 PMC5549382

[7] Kasturiratne A, Wickremasinghe AR, De Silva N, Gunawardena NK, Pathmeswaran A, Premaratna R, et al. The global burden of snakebite: A literature analysis and modelling based on regional estimates of envenoming and deaths. PLoS Medicine. 2008;5(11):1591–604. doi: 10.1371/journal.pmed.0050218 18986210 PMC2577696

[8] Tasoulis T, Isbister GK. A review and database of snake venom proteomes. Toxins. 2017;9(9):290. doi: 10.3390/toxins9090290 28927001 PMC5618223

[9] Musah Y, Ameade EPK, Attuquayefio DK, Holbech LH. Epidemiology, ecology and human perceptions of snakebites in a savanna community of northern Ghana. PLoS Neglected Tropical Diseases. 2019;13(8). doi: 10.1371/journal.pntd.0007221 31369551 PMC6692043

[10] Yakubu AS, Abdul-Mumin A, Rivera O. Evaluation of management of snake bites in a teaching hospital in Northern Ghana-a retrospective descriptive study. International Journal Of Community Medicine And Public Health. 2018;6(3). 10.18203/2394-6040.ijcmph20190573.

[11] Naik BS. “Dry bite” in venomous snakes: A review. Toxicon. 2017;133:63–7. doi: 10.1016/j.toxicon.2017.04.015 28456535

[12] Waiddyanatha S, Silva A, Siribaddana S, Isbister GK. Long-term effects of snake envenoming. Toxins. 2019;11(4):193. doi: 10.3390/toxins11040193 30935096 PMC6521273

[13] Warrell DA. Snake bite. The lancet. 2010;375(9708):77–88. 10.1016/S0140-6736(09)61754-2 20109866

[14] Golay V, Roychowdhary A, Pandey R. Spontaneous peri-nephric hematoma in a patient with acute kidney injury following Russell’s viper envenomation. Saudi Journal of Kidney Diseases and Transplantation. 2015;26(2):335. doi: 10.4103/1319-2442.152500 25758885

[15] Ranawaka UK, Lalloo DG, de Silva HJ. Neurotoxicity in snakebite—the limits of our knowledge. PLoS neglected tropical diseases. 2013;7(10):e2302. doi: 10.1371/journal.pntd.0002302 24130909 PMC3794919

[16] Chippaux J-P. Estimate of the burden of snakebites in sub-Saharan Africa: A meta-analytic approach. Toxicon. 2011;57(4):586–99. doi: 10.1016/j.toxicon.2010.12.022 21223975

[17] Visser LE, Kyei-Faried S, Belcher DW. Protocol and monitoring to improve snake bite outcomes in rural Ghana. Transactions of the Royal Society of Tropical Medicine and Hygiene. 2004;98(5):278–83. doi: 10.1016/S0035-9203(03)00065-8 15109550

[18] Yakubu AS, Abdul-Mumin A, Adam A. Clinical and demographic profile of patients with snakebite in a tertiary hospital in Ghana. Sahel Medical Journal. 2019;22(4):193.

[19] Addo V, Kokroe FA, Reindorf RL. Broad ligament haematoma following a snake bite. Ghana medical journal. 2009;43(4):181. 21327000 PMC2956370

[20] Habib AG, Kuznik A, Hamza M. Snakebite is under appreciated: appraisal of burden from West Africa. PLoS neglected tropical diseases 2015;9(9):e0004088. doi: 10.1371/journal.pntd.0004088 26398046 PMC4580425

[21] Jayawardana S, Arambepola C, Chang T, Gnanathasan A. Long-term health complications following snake envenoming. Journal of multidisciplinary healthcare. 2018;11:279. doi: 10.2147/JMDH.S126648 29983571 PMC6027691

[22] Brenes-Chacon H, Gutierrez JM, Camacho-Badilla K, Soriano-Fallas A, Ulloa-Gutierrez R, Valverde K, et al. Long-term sequelae secondary to snakebite envenoming: a single centre retrospective study in a Costa Rican paediatric hospital. BMJ Paediatrics Open. 2020;4(1):e000735. doi: 10.1136/bmjpo-2020-000735 32995568 PMC7497519

[23] Spano SJ, Vohra R, Macias F. Long-term complications of rattlesnake bites: a telephone survey from central California. Wilderness & environmental medicine. 2014;25(2):210–3. doi: 10.1016/j.wem.2013.11.004 24507436

[24] Krah E, de Kruijf J, Ragno L. Integrating traditional healers into the health care system: challenges and opportunities in rural northern Ghana. Journal of Community Health. 2018;43(1):157–63. doi: 10.1007/s10900-017-0398-4 28681282 PMC5767209

[25] Schioldann E, Mahmood MA, Kyaw MM. Why snakebite patients in Myanmar seek traditional healers despite availability of biomedical care at hospitals? Community perspectives on reasons. PLoS neglected tropical diseases. 2018;12(2):e0006299. doi: 10.1371/journal.pntd.0006299 29489824 PMC5847227

[26] Steinhorst J, Aglanu LM, Ravensbergen SJ, Dari CD, Abass KM, Mireku SO, et al. ‘The medicine is not for sale’: Practices of traditional healers in snakebite envenoming in Ghana. PLOS Neglected Tropical Diseases. 2021;15(4):e0009298. doi: 10.1371/journal.pntd.0009298 33861735 PMC8081335

[27] GSS. Ghana 2021 population and housing census: Preliminary report [Internet]. Ghana Statistical Service; September 22, 2021 [cited February 1, 2022]. Available from: https://census2021.statsghana.gov.gh/gssmain/fileUpload/reportthemelist/PRINT_COPY_VERSION_FOUR%2022ND_SEPT_AT_8_30AM.pdf.

[28] GSS. 2010 Population & housing census: National analytical report. Ghana Statistics Service, 2013 Available at https://statsghana.gov.gh/gssmain/fileUpload/pressrelease/2010_PHC_National_Analytical_Report.pdf.

[29] Pucca MB, Knudsen C, S Oliveira I, Rimbault C, A Cerni F, Wen FH, et al. Current knowledge on snake dry bites. Toxins. 2020;12(11):668. doi: 10.3390/toxins12110668 33105644 PMC7690386

[30] Üstün TB, Kostanjesek N, Chatterji S, Rehm J, (Eds). Measuring health and disability: manual for WHO Disability Assessment Schedule (WHODAS 2.0). Geneva: World Health Organization; 2010.

[31] Üstün TB, Chatterji S, Kostanjsek N, Rehm J, Kennedy C, Epping-Jordan J, et al. Developing the World Health Organization disability assessment schedule 2.0. Bulletin of the World Health Organization. 2010;88:815–23. doi: 10.2471/BLT.09.067231 21076562 PMC2971503

[32] Schlote A, Richter M, Wunderlich MT, Poppendick U, Möller C, Schwelm K, et al. WHODAS II with people after stroke and their relatives. Disability and rehabilitation. 2009;31(11):855–64. doi: 10.1080/09638280802355262 19093276

[33] Federici S, Bracalenti M, Meloni F, Luciano JV. World Health Organization disability assessment schedule 2.0: An international systematic review. Disability and rehabilitation. 2017;39(23):2347–80. doi: 10.1080/09638288.2016.1223177 27820966

[34] Kulnik ST, Nikoletou D. WHODAS 2.0 in community rehabilitation: A qualitative investigation into the validity of a generic patient-reported measure of disability. Disability and Rehabilitation. 2014;36(2):146–54. doi: 10.3109/09638288.2013.782360 23586698

[35] Wolf AC, Tate RL, Lannin NA, Middleton J, Lane-Brown A, Cameron ID. The World Health Organization Disability Assessment Scale, WHODAS II: reliability and validity in the measurement of activity and participation in a spinal cord injury population. Journal of rehabilitation medicine. 2012;44(9):747–55. doi: 10.2340/16501977-1016 22854805

[36] Stienstra Y, Dijkstra Pu Fau—Van Wezel MJ, Van Wezel Mj Fau—Van Roest MHG, Van Roest Mh Fau—Beets M, Beets M Fau—Zijlstra I, Zijlstra I Fau—Johnson RC, et al. Reliability and validity of the Buruli ulcer functional limitation score questionnaire. Am J Trop Med Hyg. 2005;72(4):449–52. 15827284

[37] Stienstra Y, Dijkstra PU, Guédénon A, Johnson RC, Ampadu EO, Mensah T, et al. Development of a questionnaire assessing Buruli ulcer–induced functional limitation. The American journal of tropical medicine and hygiene. 2004;70(3):318–22. 15031524

[38] Klis S, Kingma R, Tuah W, van der Werf TS, Stienstra Y. Clinical outcomes of Ghanaian Buruli ulcer patients who defaulted from antimicrobial therapy. Tropical Medicine & International Health. 2016;21(9):1191–6. doi: 10.1111/tmi.12745 27456068

[39] Chippaux JP. Estimate of the burden of snakebites in sub-Saharan Africa: a meta-analytic approach. Toxicon. 2011;57(4):586–99. doi: 10.1016/j.toxicon.2010.12.022 21223975

[40] Williams SS, Wijesinghe CA, Jayamanne SF, Buckley NA, Dawson AH, Lalloo DG, et al. Delayed psychological morbidity associated with snakebite envenoming. PLoS neglected tropical diseases. 2011;5(8):e1255. doi: 10.1371/journal.pntd.0001255 21829741 PMC3149015

[41] Habib ZG, Salihu AS, Hamza M, Yakasai AM, Iliyasu G, Yola IM, et al. Posttraumatic stress disorder and psycho-social impairment following snakebite in Northeastern Nigeria. The International Journal of Psychiatry in Medicine. 2020;56(2):97–115. doi: 10.1177/0091217420913400 32216497

[42] Kim KJ, Min JH, Yoo I, Kim SW, Lee J, Ryu S, et al. Negative pressure wound therapy for skin necrosis prevention after snakebite in the emergency department: A retrospective cohort study. Medicine. 2021;100(3). doi: 10.1097/MD.0000000000024290 33546055 PMC7837876

[43] Val F, Alcântara JA, Maciel Salazar GK, Farias AS, Monteiro WM, Sachett JGdA. Disability secondary to snakebites in rural Amazon: What are the impacts? Toxicon. 2020;177:S19. 10.1016/j.toxicon.2019.10.081.

[44] Eze CC, Ekeke N, Alphonsus C, Lehman L, Chukwu JN, Nwafor CC, et al. Effectiveness of self-care interventions for integrated morbidity management of skin neglected tropical diseases in Anambra State, Nigeria. BMC public health. 2021;21(1):1–15. 34563162 10.1186/s12889-021-11729-1 PMC8465703

[45] WHO. Mental health of people with neglected tropical diseases: towards a person-centred approach. 2020;(Licence: CC BY-NC-SA 3.0 IGO; https://creativecommons.org/licenses/by-nc-sa/3.0/igo).

[46] WHO. Snakebite envenoming: a strategy for prevention and control. Geneva: World Health Organization. 2019 ISBN: 978 92 4 151564 1. Licence: CC BY-NC-SA 3.0 IGO. Available from: https://apps.who.int/iris/bitstream/handle/10665/324838/9789241515641-eng.pdf?ua=1.

[47] Wijesinghe CA, Williams SS, Kasturiratne A, Dolawaththa N, Wimalaratne P, Wijewickrema B, et al. A randomized controlled trial of a brief intervention for delayed psychological effects in snakebite victims. PLoS neglected tropical diseases. 2015;9(8):e0003989. doi: 10.1371/journal.pntd.0003989 26261987 PMC4532481

